# Rotenone-induced PINK1/Parkin-mediated mitophagy: establishing a silkworm model for Parkinson’s disease potential

**DOI:** 10.3389/fnmol.2024.1359294

**Published:** 2024-04-19

**Authors:** Hantao Zhang, Jinyue Yang, Yinglu Guo, Peng Lü, Xun Gong, Keping Chen, Xiubin Li, Min Tang

**Affiliations:** ^1^School of Life Sciences, Jiangsu University, Zhenjiang, Jiangsu, China; ^2^Department of Neurology, The Second Affiliated Hospital of Shandong First Medical University, Taian, Shandong, China; ^3^Affiliated Hospital of Jiangsu University, Zhenjiang, Jiangsu, China

**Keywords:** Parkinson’s disease, silkworm, rotenone, mitophagy, animal model

## Abstract

Parkinson’s disease (PD), ranking as the second most prevalent neurodegenerative disorder globally, presents a pressing need for innovative animal models to deepen our understanding of its pathophysiology and explore potential therapeutic interventions. The development of such animal models plays a pivotal role in unraveling the complexities of PD and investigating promising treatment avenues. In this study, we employed transcriptome sequencing on BmN cells treated with 1 μg/ml rotenone, aiming to elucidate the underlying toxicological mechanisms. The investigation brought to light a significant reduction in mitochondrial membrane potential induced by rotenone, subsequently triggering mitophagy. Notably, the PTEN induced putative kinase 1 (PINK1)/Parkin pathway emerged as a key player in the cascade leading to rotenone-induced mitophagy. Furthermore, our exploration extended to silkworms exposed to 50 μg/ml rotenone, revealing distinctive motor dysfunction as well as inhibition of *Tyrosine hydroxylase (TH)* gene expression. These observed effects not only contribute valuable insights into the impact and intricate mechanisms of rotenone exposure on mitophagy but also provide robust scientific evidence supporting the utilization of rotenone in establishing a PD model in the silkworm. This comprehensive investigation not only enriches our understanding of the toxicological pathways triggered by rotenone but also highlights the potential of silkworms as a valuable model organism for PD research.

## 1 Introduction

Parkinson’s disease (PD) is the second most prevalent neurodegenerative disorder, following Alzheimer’s disease (AD) (Emamzadeh and Surguchov, 2018; Zhang et al., 2022). It can be classified into two subtypes: familial and more common sporadic (Mustapha and Mat Taib, 2021; Surguchov, 2021; Khan et al., 2023). PD is primarily caused by the degeneration of dopamine neurons in the substantia nigra and is also associated with mitochondrial dysfunction, oxidative stress, apoptosis, and inflammation (Surguchov, 2016; Van Den Berge and Ulusoy, 2022). A myriad of motor symptoms, including tremor, rigidity, postural abnormalities, bradykinesia, and akinesia, are experienced by people with PD (Salari and Bagheri, 2019). Since current therapies only alleviate motor symptoms without curing the disease, and its incidence is constantly increasing, extensive research is necessary to fully understand the pathogenesis of the disease (Taguchi et al., 2020). This will facilitate the development of early diagnostic methods and therapeutic strategies to impede disease progression (Giuliano et al., 2021). Animal models play a crucial role in these studies, as obtaining human brains is challenging, and these models can accurately simulate many aspects of disease characteristics (Ibarra-Gutiérrez et al., 2023).

Current models for PD in animals can be classified into two main types: poison models and genetic models (Chia et al., 2020). The use of neurotoxins, such as rotenone, is the basis for the establishment of poison models (Zeng et al., 2018). Rotenone belongs to the class of isoflavones and has the ability to readily cross the blood–brain barrier and cell membranes due to its lipophilicity (Pingale and Gupta, 2020). When rotenone enters dopaminergic neurons, its primary effect is to inhibit the NADH-ubiquinone oxidoreductase (complex I) that allows free electrons to reduce oxygen to reactive oxygen species (ROS) (Radad et al., 2019). The increased production of ROS, coupled with the decreased activity of antioxidant enzymes under the influence of rotenone, results in oxidative stress, which ultimately cause the death of dopaminergic neurons (Innos and Hickey, 2021). The loss of the dopamine neurons results in the manifestation of the standard behavioral symptoms of PD (Kin et al., 2019).

Currently, a variety of vertebrate animals, including monkeys, mice, and zebrafish, as well as some invertebrate animals such as *Drosophila* and *Caenorhabditis elegans*, have been used by researchers to model PD (Heikkila et al., 1985; Ferrante et al., 1997; Betarbet et al., 2000; Nass et al., 2002; Feng et al., 2014; Shukla et al., 2014; Kikuchi et al., 2017). Silkworms (*Bombyx mori*) may also have the potential to serve as an experimental model for PD, since there are 8,469 human homologous genes in its genome, which are included in pathways related to neurodegenerative diseases and oxidative stress (Tabunoki et al., 2016). By administering 6-hydroxydopamine (6-OHDA) to silkworms chronically, Zhu et al. (2022) induced symptoms in silkworms that were comparable to those seen in humans with PD, such as movement disorders, loss of dopaminergic neurons, and reduced dopamine levels. Later, Song et al. (2022) established a model of PD by feeding silkworms with 1-methyl-4-phenyl-1,2,3,6-tetrahydropyridine (MPTP). The model exhibited behavioral impairment, reduced dopamine metabolism, and oxidative stress (Song et al., 2022). However, rotenone has not yet been utilized to develop a PD model in the silkworm. Investigation into the effects of rotenone on silkworms, the method of administration and dose of rotenone that can be used to reproduce the pathological symptoms of PD in silkworms, as well as its side effects and underlying molecular mechanisms, is necessary.

In this study, BmN cells from silkworm ovarian tissue were used to study the mechanism of rotenone toxicity. It was indicated that rotenone decreased cell viability and induced mitophagy. In order to gain a better understanding of the mechanisms involved in rotenone-induced mitophagy, we analyzed the changes in gene expression levels in BmN cells treated with rotenone. The results of the *in vitro* experiments, combined with the transcriptome analysis, showed for the first time that rotenone activated the PINK1/Parkin pathway and thus caused mitophagy in silkworms. In addition, we established a silkworm-based PD model using rotenone. This model was then used to investigate locomotion impairment and dopamine synthesis. We hope that our work would provide scientific evidence for the mechanism of toxicity of rotenone and its application in constructing animal models of PD.

## 2 Materials and methods

### 2.1 Cell culture and cell viability assay

The silkworm cell line BmN originated from ovarian was used in this study. BmN cells were maintained in our lab, culturing in TC-100 insect medium supplemented with 10% fetal bovine serum (Gibco/Thermo Fisher Scientific, Waltham, MA, USA) and 1% antibiotic–antimycotic mixture at 27°C.

To determine the required concentration of rotenone, cell viability was measured using Cell Counting Kit-8 (CCK-8) (MedChemExpress, China). Briefly, BmN cells were subcultured in 24-well plates at a seeding density of 5 × 10^4^ cells per well and treated with rotenone (0.05, 0.5, 1, 10, or 50 μg/ml) for 2 days. Rotenone was diluted in dimethyl sulfoxide (DMSO). After treatment, 10 μl of CCK-8 solution was added to each well of the plates, and the plates were kept in the incubator at 37°C for 2 h. The absorbance at 450 nm was measured using a microplate reader. Data were expressed as mean ± SD from six independent experiments.

### 2.2 *In vitro* experiment

To comprehensively evaluate the impact of rotenone on BmN cells, we categorized the cells into distinct groups for detailed investigation. Group I, designated as the Control group, involved the incubation of BmN cells in a medium supplemented with DMSO for a period of 2 days. Meanwhile, in Group II, labeled as the Rotenone group, BmN cells were exposed to a medium containing freshly diluted rotenone at a concentration of 1 μg/ml for a duration of 2 days. It is worth noting that rotenone was meticulously prepared and diluted in DMSO to ensure optimal experimental conditions.

### 2.3 Detection of mitochondrial membrane potential

Detection of the mitochondrial membrane potential (MMP) was performed using a HCS Mitochondrial Health Kit (Molecular Probes™, Invitrogen) according to the manufacturer’s instructions. After drug treatment, 50 μl of cell staining solution containing 10.5 μl of the MitoHealth stain solution and 2.1 μl of Image-iT^®^ DEAD Green™ viability stain was added to each well of the plates. After 30 min of incubation at 27°C, the medium was removed and the cells were incubated with 100 μl of counterstain/fixation solution containing 6 μl of Hoechst 33342 for 15 min. Finally, the counterstain/fixation solution was removed and the wells were rinsed with 100 μl of PBS. A fluorescent microscope was used to scan the plates.

### 2.4 Immunofluorescence staining

To study the formation of mitolysosome, mitochondria and lysosomes in BmN cells were labeled using MitoTracker^®^ Red CMXRos (Thermo Fisher Scientific) and LysoTracker^®^ Green DND-26 (Thermo Fisher Scientific), respectively, with reference to the manufacturer’s instructions. After drug treatment, the medium was removed from the plates and pre-warmed staining solution containing MitoTracker^®^ Red probe and LysoTracker^®^ Green probe was added, followed by 3 h of incubation. Co-localization of mitochondria and lysosomes were observed by a fluorescent microscope (LSCM system, LSM800, Zeiss, Germany) in the dark.

### 2.5 Transcriptome sequencing

Total RNA was extracted from cultured cells using TRIzol reagent kit (Invitrogen, Carlsbad, CA, USA) according to the manufacturer’s instructions. RNA quality was assessed on an Agilent 2100 Bioanalyzer (Agilent Technologies, Palo Alto, CA, USA) and checked using RNase free agarose gel electrophoresis. Afterward, mRNA was enriched by Oligo(dT) beads, fragmented into short fragments using a fragmentation buffer, and reversely transcribed into cDNA by using NEBNext Ultra RNA Library Prep Kit for Illumina (New England Biolabs, Ipswich, MA, USA). The purified double-stranded cDNA fragments were subjected to end repair, a base addition, and ligation of Illumina sequencing adapters. The ligation reaction was purified with the AMPure XP Beads (1.0X), followed by polymerase chain reaction (PCR) amplified. The resulting cDNA library was sequenced on the Illumina Novaseq6000.

### 2.6 Transcriptome profiling analysis

After sequencing, raw reads were preprocessed using fastp (v.0.23.4) by removing adaptor sequences and filtering low-quality reads (Chen, 2023). FastQC (v.0.12.1) was used for quality control before and after preprocessing (Patel and Jain, 2012). The trimmed and filtered high-quality clean reads were aligned to the *B. mori* genome (GCF_014905235.1) by HISAT2 (v.2.2.1) with default parameters (Kim et al., 2019), and the gene expression levels were estimated using featureCounts (v.2.0.3) (Liao Y. et al., 2014). Subsequently, a differentially expressed analysis of silkworms involved in rotenone and DMSO was conducted via DESeq2 (v.1.38.3) with default parameters (Love et al., 2014). To address the high rate of false positives resulting from multiple testing, the Benjamini–Hochberg method was used to adjust the *p*-value. Genes with adjusted *p*-values < 0.05 and an absolute log2(Fold Change) > 1 were recognized as differentially expressed genes (DEGs). These DEGs were subjected to the Gene Ontology (GO) function analysis and KEGG pathway enrichment using clusterProfiler (v.4.6.2) with a Benjamini–Hochberg corrected *p*-value < 0.05 (Wu et al., 2021). The analysis results were presented in the form of bubble and hierarchical bar charts.

### 2.7 Behavioral study and brain tissue preparation

**FIGURE 1:**
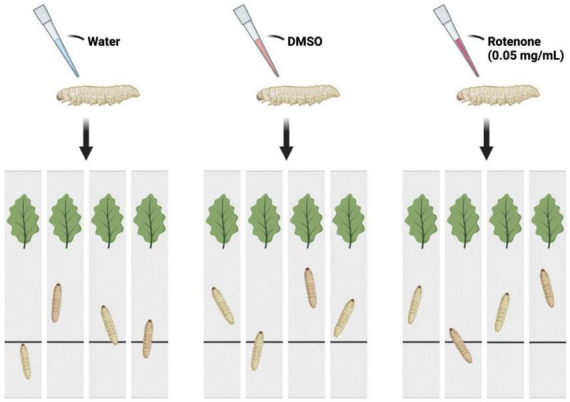
The schematic diagram of the motion testing. The larvae were separated into three groups and administered drops of water, DMSO, and rotenone on their heads. After a duration of 24 h, each was placed at a starting point on a strip of cardboard. The time taken for each larva to reach mulberry leaves situated at the opposite end of the cardboard strip was recorded.

The *B. mori* (strain: 306) was obtained from the Institute of Life Sciences, Jiangsu University. The silkworm larvae were reared with fresh mulberry leaves under the condition of 25 ± 2°C and 12 h light/dark cycle. On the first day of fifth instar, 90 larvae were randomly divided into three groups: Control group, DMSO group, and Rotenone group (*n* = 30/condition). Drops of water, DMSO, and 50 μg/ml rotenone were applied to the heads of the silkworms in Control group, DMSO group, and Rotenone group, respectively. After 24 h, 4 larvae were randomly selected from each group to determine the motor function. Another treatment was performed on 15 selected silkworms from each group, following the same procedure as the first round. After 24 h, four individuals were selected from each group for motion testing. Figure 1 showed the method used to assess the motor function of silkworms. Each silkworm was placed on a strip of cardboard and the time it took them to move from their starting point to the mulberry leaves was recorded. In addition, six individuals were selected from each of DMSO group and Rotenone group for brain tissue collection. The brain tissues were washed twice with sterile PBS, packed into 1.5 ml EP tubes, frozen with liquid nitrogen, and stored in a refrigerator at −80°C.

### 2.8 Quantitative real-time PCR

Total RNA was isolated with UNlQ-10 Column TRIzol Total RNA Isolation Kit (Sangon Biotechnology, Shanghai, China) and reversely transcribed into cDNA using HiScript III RT SuperMix with gDNA wiper (Vazyme, Nanjing, China). Gene expression levels were quantified by an ABI 7,500 Sequence Detector (Applied Biosystem, Carlsbad, CA, USA) with the AceQ qPCR SYBR Green Master Mix (Vazyme Biotechnology, Nanjing, China). Primer sequences synthesized by GENEWIZ were shown in Table 1. Reactions were carried out with 2.8 μl reverse transcribed RNA and gene-specific primers in a total volume of 20 μl. PCR conditions consisted of an initial incubation step of 2 min at 50°C and a heat activation step of 10 min at 95°C. This was followed by 40 cycles of denaturation at 95°C for 10 s and annealing and extension at 60°C for 30 s.

**TABLE 1:** The primer sequences for RT-qPCR used in this study.

Gene name	Forward primer (5′-3′)	Reverse primer (5′-3′)
*AMBRA1*	CGACTCGGACAGTGATCGTT	CATTAAACGGTCGGTCGGGA
*ATF4*	TCGTGTCGTGCTATGGTGTC	CTCTGCGGCGGTGTTAAATG
*ATG8*	CTAGGCTTGGAGACCTCGAC	AGAGAGACCCCATTGTTGCAG
LOC692779	AGTGGCGAAGTTTCCAGAGG	CGAATATTTGCATTGTGTTGCTCC
*MFN2*	TATGGAGCAGGTACGCACAC	GGCTTCCTTGGCGGATATGA
*p62*	AAGAAGTGGAGCGTCCCAAG	AGTTGCTGGATTTGGGGGAG
*Parkin*	GAAGATGACACGAAAGACGATG	CTCAAGCTCAGTTTGTCTTTCC
*TBK1*	GCCGAAATTAAGCTGGTCGC	TGGCTTCAATCTTCACCGCT
*USP30*	GGGTTTGAAGCTAAACGGTGG	GCTGAGTGTCCTGTCTCTCC
*TH*	TTCAGGACTGAACACAAACTCT	GGTTCTGCGGTTCTTTATCATC

## 3 Results

### 3.1 Rotenone reduced cell viability

**FIGURE 2:**
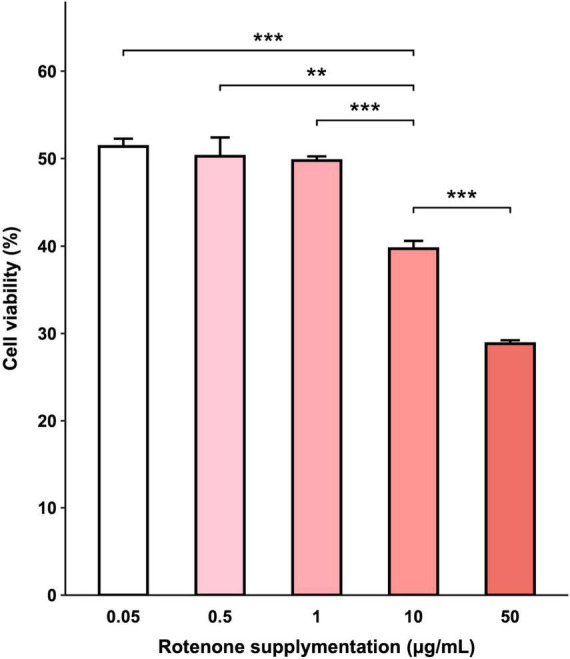
Effects of rotenone on BmN cell viability. CCK-8 assay was used to determine cell viability after being treated with different concentrations of rotenone. Each value represents the mean of six independent experiments ± SEM. The *p*-value was determined by Student’s *t*-test (**p < 0.01, ***p < 0.001).

The viability of BmN cells treated with increasing concentrations of rotenone was examined by CCK-8 assay and the results indicated that exposure to rotenone caused a dose-dependent decrease in cell viability. As shown in Figure 2, increasing the concentration of rotenone from 0.05 to 1 μg/ml did not result in a significant change in cell viability. However, when the concentration was further increased to 10 μg/ml, a significant decrease in cell viability was observed in comparison with the cells treated with 0.05, 0.5, and 1 μg/ml rotenone. After 48-h rotenone treatment, the cell viability was 39.72% in the 10 μg/ml rotenone-exposed group, while only 28.83% was left in the highest concentration group (50 μg/ml), implying that rotenone can exert an antiproliferative effect. To further investigate the adverse effects of rotenone on mitochondrial function and mitophagy, BmN cells were treated with 1 μg/ml rotenone.

### 3.2 Rotenone induced mitochondrial dysfunction and mitophagy

**FIGURE 3:**
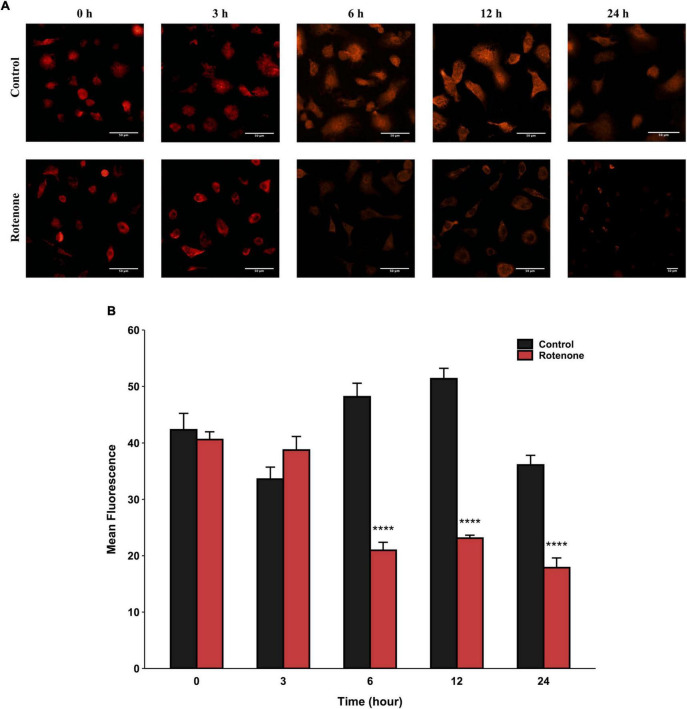
Effects of rotenone on MMP of BmN cells. Representative images **(A)** and quantitative analysis **(B)** of the mean fluorescence intensity of MitoHealth in DMSO-treated and rotenone-treated BmN cells at the six different time-points. Each value represents the mean of three independent experiments ± SEM. The *p*-value was determined by Student’s *t*-test (****p < 0.0001).

**FIGURE 4:**
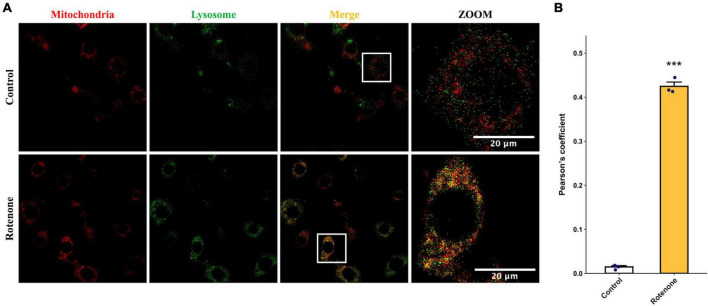
Effects of rotenone on mitophagy of BmN cells. **(A)** Assessment of the effects of rotenone on mitophagy using MitoTracker Red and LysoTracker Green. Representative images are presented, and the yellow areas indicate mitochondria-containing autolysosomes. Scale bars, 20 μm. **(B)** Quantification of colocalization of MitoTracker Red and LysoTracker Green in DMSO-treated and rotenone-treated BmN cells were measured by Pearson correlation. Each value represents the mean of three independent experiments ± SEM. The *p*-value was determined by Student’s *t*-test (***p < 0.001).

The cytotoxic effect of rotenone is known as the inhibition of mitochondrial complex I, which may lead to mitochondrial dysfunction and damage (Pravdic et al., 2012; Basit et al., 2017; Heinz et al., 2017; Morales-Martinez et al., 2022). Since the MMP is a key indicator for evaluating mitochondrial function, we measured the changes in MMP in BmN cells treated with DMSO or rotenone using HCS Mitochondrial Health Kit (Sakamuru et al., 2016). As shown in Figure 3A, the red fluorescence of the MitoHealth stain in the Rotenone group was significantly lower than that of the Control group after 6, 12, and 24 h of rotenone treatment, suggesting a decrease in MMP in rotenone-treated BmN cells (Figure 3B). Mitochondrial dysfunction is an essential precursor event that triggers mitochondrial autophagy (Lu et al., 2023). To maintain mitochondrial as well as cellular homeostasis, cells use the mechanism of mitochondrial autophagy to deliver damaged mitochondria to lysosomes by autophagosomes for degradation (Xu et al., 2020). Therefore, to investigate the effect of rotenone on mitophagy levels, we labeled mitochondria and lysosomes with MitoTracker^®^ Red probes and LysoTracker^®^ Green probes, respectively. The formation of mitochondria-containing autolysosomes was indicated by yellow fluorescence. As shown in Figure 4A, the appearance of mitochondria-containing autolysosomes was clearly observed in cells after 12 h of rotenone treatment, whereas it was not observed in cells following 12 h of DMSO treatment. Furthermore, we employed Pearson’s correlation coefficient to estimate the colocalization of mitochondria and autolysosomes. Pearson’s *R* value between MitoTracker^®^ Red and LysoTracker^®^ Green increased from 0.01 in DMSO-treated cells to 0.42 in rotenone-treated cells (Figure 4B). Together, the images and correlation analyses consistently indicated increased mitophagy levels after rotenone treatment.

### 3.3 Collection and analysis of transcriptome data

**FIGURE 5:**
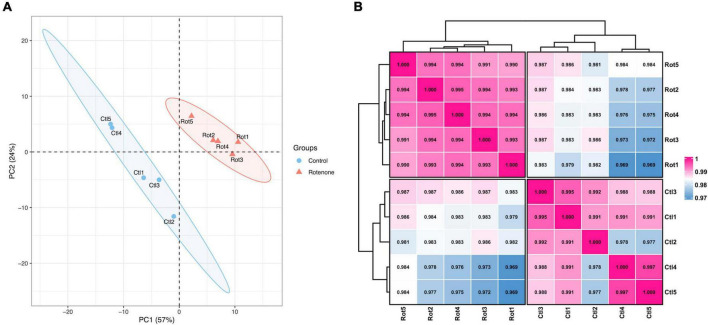
Overview of transcriptome sequencing data. **(A)** Principal component analysis of samples. **(B)** Pearson correlation analysis of samples. The number is the correlation coefficient between the two samples.

In order to investigate the effect of rotenone on gene expression in BmN cells, high-throughput transcriptome sequencing was performed for the DMSO-treated group (Control group) and the rotenone-treated group (Rotenone group), with five replicates for each group. After preprocessing, 40,493,214, 39,872,296, 37,184,136, 42,461,534, and 40,465,356 clean reads were obtained from the Control group and 37,397,810, 39,760,342, 38,444,916, 42,751,288, and 41,371,804 clean reads were obtained from the Rotenone group. For the Control group, the GC content and Q30 percentage of the clean data ranged from 46.59% to 47.67% and from 92.75% to 93.65%, and a mean of 76.58% of clean reads was mapped to the *B. mori* genome. Besides, for the Rotenone group, the GC content and Q30 percentage of the clean data ranged from 41.36% to 48.50% and from 92.52% to 93.14%, and a mean of 75.45% of clean reads was mapped to the *B. mori* genome (Table 2). The results of principal component analysis (PCA) and Pearson correlation analysis demonstrated that all 10 samples were clearly divided into two clusters, and that there were significant differences between these two clusters (Figure 5).

**TABLE 2:** Summary of the sequencing data from RNA sequencing.

Samples	Clean reads (%)	Q20 (%)	Q30 (%)	GC content (%)	Overall alignment rate (%)	Unique mapping rate (%)	Multiple mapping rate (%)	Notes
Ctl1	40,493,214	97.39	93.15	47.32	76.00	69.66	6.35	Replicate 1
Ctl2	39,872,296	97.62	93.65	47.67	76.69	69.62	7.08	Replicate 2
Ctl3	37,184,136	97.22	92.91	47.34	76.81	70.21	6.60	Replicate 3
Ctl4	42,461,534	97.47	93.38	46.72	76.97	71.41	5.57	Replicate 4
Ctl5	40,465,356	97.16	92.75	46.59	76.42	70.84	5.57	Replicate 5
Rot1	37,397,810	97.07	92.52	48.50	75.23	68.24	6.99	Replicate 1
Rot2	39,760,342	97.35	93.12	47.90	76.99	69.76	7.22	Replicate 2
Rot3	38,444,916	97.32	93.14	48.14	75.83	67.83	8.00	Replicate 3
Rot4	42,751,288	97.16	92.74	48.16	74.87	67.68	7.20	Replicate 4
Rot5	41,371,804	97.26	92.99	41.36	74.33	67.77	6.56	Replicate 5

**FIGURE 6:**
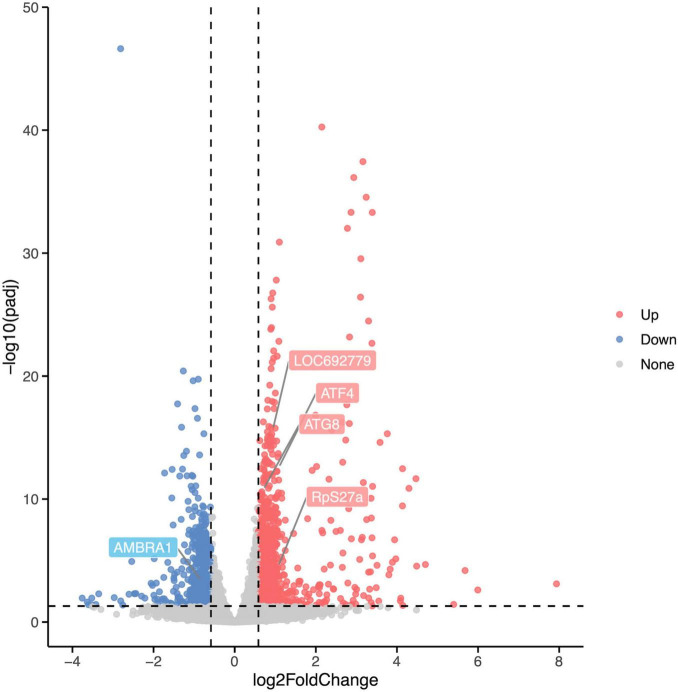
Differential expression analysis of Control groups and Rotenone groups. A total of 1,454 DEGs were identified in Rotenone groups, including 877 upregulated (log_2_FC < –log_2_1.5, corrected *p*-value < 0.05) and 577 downregulated (log_2_FC > log_2_1.5, corrected *p*-value < 0.05). Compared to the Control groups, the Rotenone groups showed a significant upregulation in the expression of LOC692779, *ATF4*, *ATG8*, and *RpS27a* and a significant downregulation in the expression of *AMBRA1*.

Differentially expressed analysis was performed using the R package DESeq2 to identify DEGs between the Rotenone group and the Control group to investigate the impact of rotenone on gene expression levels. A total of 1,454 genes passed the fold change (FC) and corrected *p*-value filters (absolute log_2_FC > log_2_1.5, corrected *p*-value < 0.05), including 877 significantly upregulated and 577 downregulated in the Rotenone group compared to the Control group, as represented in a volcano plot (Figure 6).

### 3.4 GO and KEGG enrichment analysis of DEGs induced by rotenone

To elucidate the potential functions and related biological processes of the 1,454 DEGs, we performed the GO function analysis and KEGG pathway enrichment on these genes using the R package clusterProfiler. The results with corrected *p*-values < 0.05 were considered statistically significant.

**FIGURE 7:**
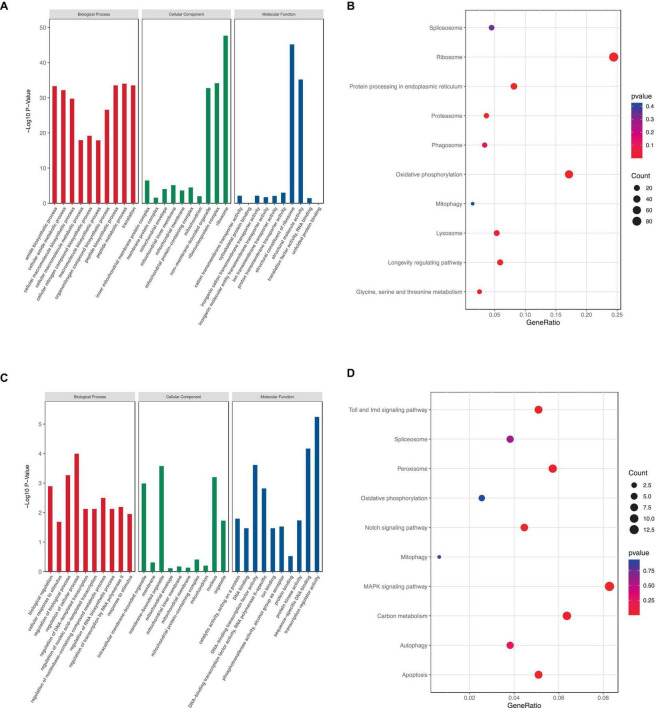
KEGG and GO analysis of DEGs. **(A,C)** GO term enrichment analysis of up- and downregulated DEGs. **(B,D)** KEGG pathway enrichment analysis of up- and downregulated DEGs.

The number of GO terms in the biological process (BP), cellular component (CC), and molecular function (MF) categories obtained from the GO enrichment analysis for upregulated DEGs were 51, 2, and 37, respectively. In downregulated DEGs, the number of GO terms in the BP, CC, and MF categories were 2, 3, and 4, respectively. We chose the primary BP, CC, and MF terms from each analysis result for visualization (Figures 7A, C). The enrichment of “mitochondrion,” “mitochondrial membrane,” “mitochondrial inner membrane,” “mitochondrial protein-containing complex,” “inner mitochondrial membrane protein complex,” and “mitochondrial envelope” suggested that rotenone may impact mitochondria in BmN cells.

Furthermore, KEGG pathway enrichment analysis showed that a total of 7 KEGG pathways were significantly enriched. The upregulated DEGs were mainly enriched in six pathways, including “Ribosome,” “Protein processing in endoplasmic reticulum,” “Proteasome,” “Oxidative phosphorylation,” “mitophagy,” and “Lysosome,” whereas the downregulated DEGs were mainly enriched in eight pathways: “Toll and Imd signaling pathway,” “Peroxisome,” “Notch signaling pathway,” “Mitophagy,” “MAPK signaling pathway,” “Carbon metabolism,” “Autophagy,” and “Apoptosis” (Figures 7B, D). These results indicated that rotenone’s toxicity may be related to its effects on ATP synthesis, removal of dysfunctional mitochondria or other intracellular structures, and cell death.

### 3.5 Rotenone activated PTEN induced putative kinase 1/Parkin pathway

**FIGURE 8:**
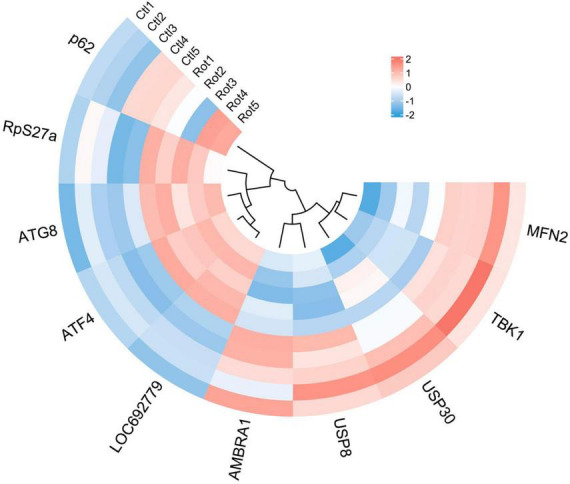
Heatmap of genes in the PINK1/Parkin signaling pathway.

**FIGURE 9:**
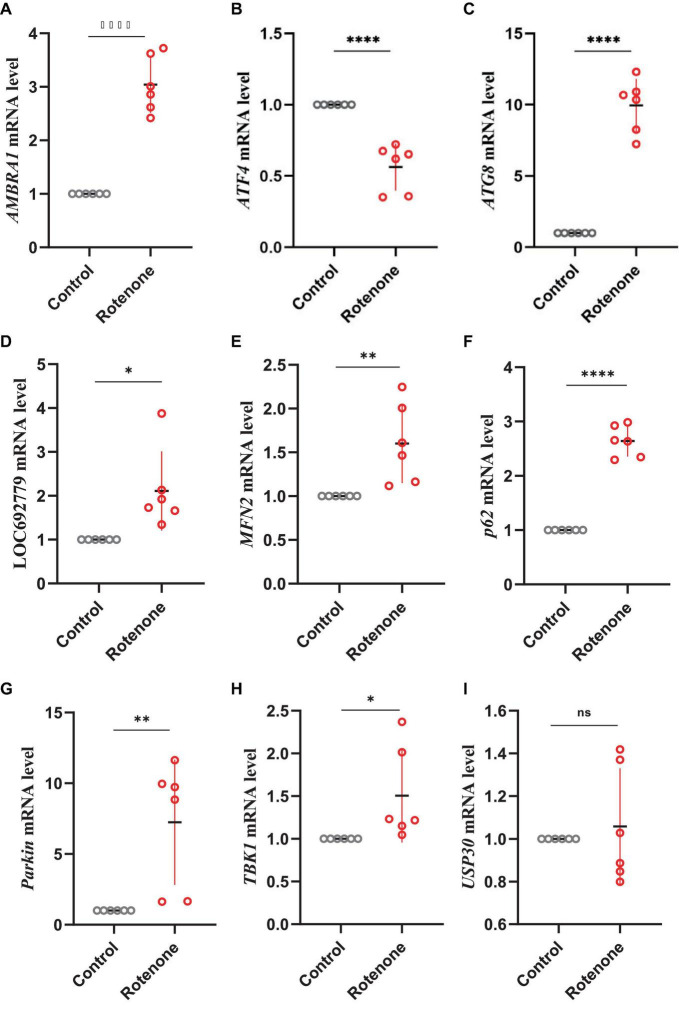
Effects of rotenone on the expression of genes in the PINK1/Parkin signaling pathway. Quantitative measurement of panel **(A)** AMBRA1, **(B)** ATF4, **(C)** ATG8, **(D)** LOC692779, **(E)** MFN2, **(F)** p62, **(G)** Parkin, **(H)** TBK1, and **(I)** USP30 mRNA in the BmN cells was carried out after treatment with DMSO and rotenone. Results are displayed as mean ± SD (*n* = 6/condition). The *p*-value was determined by Student’s *t*-test (*p < 0.05, **p < 0.01, ****p < 0.0001).

PTEN induced putative kinase 1 (PINK1)/Parkin pathway may be involved in the mitophagy induced by rotenone. Hence, we analyzed the expression levels of genes that encode the primary components in the PINK1/Parkin-mediated mitophagy. The transcriptome analysis and quantitative real-time PCR (qRT-PCR) results showed that in Rotenone groups, the expression of RpS27a, LOC692779, and ATG8 was significantly elevated, while there was no difference in USP30 expression (Figures 6, 8, 9). Furthermore, the genes MFN2, Parkin, USP8, p62, and TBK1 were not identified as DEGs. However, the results of RT-qPCR showed a significant increase in the mRNA levels of MFN2, Parkin, p62, and TBK1 in Rotenone groups (Figures 6, 8, 9). In addition, the differentially expressed analysis revealed significant downregulation of *AMBRA1* and upregulation of *ATF4* in Rotenone groups, while RT-qPCR results were the opposite (Figures 6, 8, 9). These findings indicated that rotenone may induce PINK1/Parkin-mediated mitophagy.

### 3.6 Rotenone impaired the movement of silkworms

**FIGURE 10:**
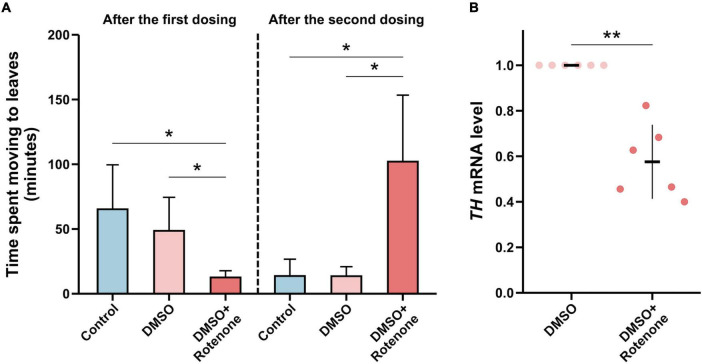
Evaluation of the silkworm model of PD constructed using rotenone. **(A)** A comparison between the motor function of silkworms after the first and second treatments with water, DMSO, and rotenone (*n* = 4/condition). The motor function was determined by measuring the time it took silkworms to move to mulberry leaves. **(B)** A comparison of *TH* expression levels in the brain tissues of silkworms receiving two DMSO treatments or rotenone treatments (*n* = 6/condition). Each value was represents as the mean with SD. The *p*-value was determined by Wilcoxon–Mann–Whitney test and Student’s *t*-test, respectively (*p < 0.05, **p < 0.01).

To study the effect of rotenone on the movement of silkworms, we placed the silkworms treated with water, DMSO, or rotenone at starting points on cardboard boards with mulberry leaves at the opposite ends. We then measured the time it took the silkworms to crawl from the starting point to the mulberry leaves to assess their mobility. Twenty-four hours after the first treatment, the silkworms with rotenone dropped on their heads moved significantly faster than those with water or DMSO, which may indicated that the rotenone treatment had activated their survival instinct, causing them to crawl toward the mulberry leaf (Figure 10A). However, 24 h after the second treatment, the movement speed of the rotenone-treated silkworms was significantly slower than that of the water- or DMSO-treated silkworms, suggesting that rotenone may induce motor dysfunction in the silkworms (Figure 10A). Dopamine is a neurotransmitter that regulates motion (Bertoldi, 2014). To investigate whether rotenone also affected dopamine synthesis, we analyzed the expression of *Tyrosine hydroxylase* (*TH*). As shown in Figure 10B, the level of *TH* expression was significantly downregulated in Rotenone groups. Since TH is the rate-limiting enzyme in dopamine synthesis, suppression of *TH* expression may disturb dopamine synthesis (Daubner et al., 2011). This could partly explain why rotenone impaired the locomotion of the silkworms. Together, these results suggested that the phenotypes of the silkworms treated with rotenone shared similarities with some symptoms of PD in humans. This indicated that rotenone showed promise for use in establishing a silkworm-based PD model.

## 4 Discussion

Rotenone functions both as a selective piscicide and a broad-spectrum insecticide (Radad et al., 2019). Additionally, rotenone is employed in developing PD animal models. Researchers have used rotenone to reproduce behavioral and neuropathological parkinsonian features in animals such as *Lymnaea stagnalis*, *Drosophila*, zebrafish, and rats, but no such studies have been conducted in silkworms (Betarbet et al., 2000; Vehovszky et al., 2007; Liao J. et al., 2014; Chen et al., 2015; Basil et al., 2017; Maasz et al., 2017; Zhang et al., 2017). Therefore, this study aimed to evaluate the possibility of utilizing rotenone in constructing a PD model in silkworms. To achieve this goal, we examined the impact of rotenone on silkworms in detail.

Rotenone induced mitochondrial depolarization. Previous studies have shown that rotenone prevented the transfer of electrons from complex I to ubiquinone (Li et al., 2003). As a result, electrons escaped from complex I, ultimately reducing oxygen to ROS (Nolfi-Donegan et al., 2020). The generation of ROS was found to stimulate the endoplasmic reticulum (ER)-calcium release channels, causing the release of calcium stored in the ER into the cytoplasm (Zhong et al., 2018). The calcium then entered the mitochondria, leading to the loss of MMP (Duchen, 2000). In the current study, we found that the upregulated DEGs in rotenone-treated BmN cells were significantly enriched in GO terms and KEGG pathways related to oxidative phosphorylation. Activation of the oxidative phosphorylation could potentially serve as a mechanism to counteract the blockade of ATP synthesis caused by rotenone-induced electron transfer inhibition. Furthermore, the upregulated DEGs were significantly enriched in the longevity regulating pathway, including genes encoding superoxide dismutase (SOD), which may indirectly indicate increased levels of ROS in rotenone-affected cells. After 6 h of treatment with rotenone, the mitochondrial depolarization was observed.

**FIGURE 11:**
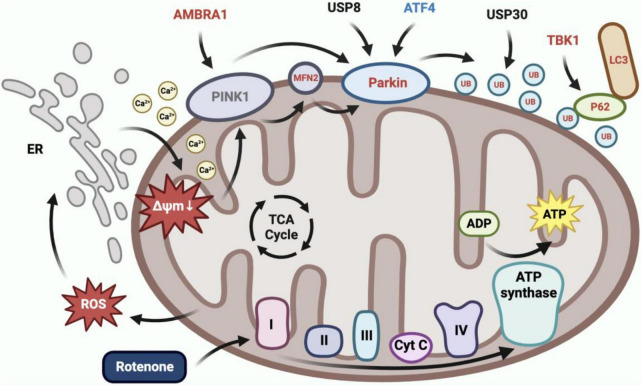
Rotenone induced PINK1/Parkin-mediated mitophagy. Inhibition of complex I in the electron transport chain by rotenone would increase ROS production, which caused calcium to flow from the ER into the cytoplasm and subsequently into mitochondria, ultimately leading to mitochondrial depolarization. BmN cells utilized the PINK1/Parkin-mediated mitophagy to eliminate depolarized mitochondria. The RT-qPCR results demonstrated a significant upregulation in the expression of *AMBRA1*, *MFN2*, *Parkin*, *RpS27a*, LOC692779, *p62*, *TBK1*, and *ATG8*, and a significant downregulation in the expression of *ATF4* in Rotenone groups.

Rotenone elicited mitophagy (Figure 11). In our present study, we observed the co-localization of mitochondria and lysosomes in rotenone-treated cells, indicating the occurrence of mitophagy. This may be related to the activation of the PINK1/Parkin signaling pathway, as the expression of *MFN2*, *Parkin*, *RpS27a*, LOC692779, *p62*, *TBK1*, and *ATG8* in this pathway was upregulated. Parkin is a critical component of the PINK1/Parkin pathway, and MFN2 was found to assist in the recruitment of Parkin to damaged mitochondria (Bhatia et al., 2019; Barazzuol et al., 2020). Upon activation by PINK1, Parkin can coordinate the formation of ubiquitin chains on a large number of OMM proteins (Ashrafi and Schwarz, 2013). P62 can recognize ubiquitinated OMM proteins and interact with ATG8 to anchor damaged mitochondria into autophagy (Shin et al., 2020). TBK1 mediated the p62 phosphorylation, which may promote mitophagy (Xiao et al., 2022). The impact of rotenone on the PINK1/Parkin-mediated mitophagy has also been reported in previous studies. Park and Koh (2020) and Wang et al. (2021) discovered that rotenone treatment significantly enhanced the expression of LC3II and p62 in SH-SY5Y cells. In addition, Heo et al. (2022) found increased levels of ubiquitin and LC3 expression in rotenone-exposed porcine oocytes. These findings aligned with our results. However, our results are contradicted by the decreased expression levels of p62 observed by Peng et al. (2019) in PC12 cells treated with rotenone. Therefore, further research is necessary to investigate the mechanisms through which rotenone impacts the expression of genes in the PINK1/Parkin pathway.

Rotenone induced PD-like motor symptoms in silkworms. In the current study, the movement speed of the silkworms that received both the initial and subsequent 50 μg/ml rotenone treatments and the expression levels of *TH* in their brain tissue were markedly decreased. Locomotion deficiencies are comparably apparent in other animal models of PD that have been established with rotenone. Cannon et al. (2009) found that male Lewis rats developed bradykinesia, postural instability, and/or rigidity after 60 days of intraperitoneal rotenone injections. In addition, Vehovszky et al. (2007) discovered that chronic treatment with rotenone inhibited spontaneous locomotion and feeding in *L. stagnalis*. In silkworm models for PD constructed with other neurotoxins such as 6-OHDA and MPTP, downregulation of dopamine levels and *TH* expression were observed (Song et al., 2022; Zhu et al., 2022). We hypothesized that oxidative stress was closely associated with the manifestation of PD-like characteristics in silkworms treated with rotenone. Rotenone easily permeated cell membranes and had an inhibitory impact on the activity of complex I (Richardson et al., 2019). Inhibiting complex I activity in dopaminergic neurons would increase the availability of electrons to generate ROS (Bisbal and Sanchez, 2019). Although the cells were able to eliminate dysfunctional mitochondria through mitophagy, thereby partially alleviating the toxic effects of rotenone, the excess of ROS would lead to oxidative stress (Brennan-Minnella et al., 2016). Oxidative stress may cause the deterioration of dopaminergic neurons, contributing to the pathogenesis of PD (Sanders and Timothy Greenamyre, 2013). In conclusion, the results of the behavioral studies in silkworms indicated that rotenone may be a suitable tool for the development of PD models in silkworms; however, further research is needed to investigate the feasibility of inducing other PD-like symptoms in silkworms with rotenone and the associations between rotenone exposure and these symptoms.

## 5 Conclusion and perspective

In summary, the findings from our behavioral studies in silkworms indicate that rotenone holds promise as a viable tool for establishing PD models in this unique species. The observed reduction in movement speed, locomotion deficiencies, and *TH* expression levels in rotenone-treated silkworms aligns with characteristic PD motor symptoms. This suggests that silkworms could potentially serve as a valuable model organism for investigating specific aspects of PD pathology.

Nonetheless, the full potential of rotenone in recapitulating the diverse spectrum of PD-like symptoms in silkworms remains to be explored. Future investigations should delve deeper into the multifaceted nature of PD by considering a broader range of symptoms. Understanding the nuanced associations between rotenone exposure and various PD manifestations will provide a comprehensive view of the modeling capabilities of silkworms in the context of PD research. Moreover, the molecular underpinnings of rotenone-induced PD-like symptoms in silkworms warrant in-depth exploration. Investigating the intricate mechanisms involved in oxidative stress, mitophagy, and neurodegeneration will not only enhance our understanding of PD pathogenesis but also contribute to refining the silkworm model. This, in turn, could lead to the development of a more nuanced and clinically relevant representation of PD in silkworms. In essence, while the initial behavioral outcomes are promising, the full potential of the silkworm model for PD research hinges on unraveling the complexities associated with rotenone exposure. Further studies are essential to unlock the complete spectrum of PD-like symptoms in silkworms and to establish this model as a robust platform for advancing our understanding of PD.

## Data availability statement

The raw sequence data reported in this paper have been deposited in the Genome Sequence Archive (Genomics, Proteomics & Bioinformatics 2021) in National Genomics Data Center (Nucleic Acids Res 2022), China National Center for Bioinformation/Beijing Institute of Genomics, Chinese Academy of Sciences (GSA: CRA015701) that are publicly accessible at https://ngdc.cncb.ac.cn/gsa.

## Ethics statement

Ethical approval was not required for the study involving animals in accordance with the local legislation and institutional requirements because the animal used here was an invertebrate.

## Author contributions

HZ: Data curation, Formal analysis, Investigation, Software, Writing – original draft. JY: Funding acquisition, Investigation, Resources, Writing – review & editing. YG: Validation, Visualization, Writing – review & editing. PL: Project administration, Resources, Writing – review & editing. XG: Funding acquisition, Resources, Writing – review & editing. KC: Funding acquisition, Project administration, Resources, Writing – review & editing. XL: Funding acquisition, Writing – review & editing. MT: Supervision, Writing – review & editing.
